# Alloparental care in glassfrogs: males care for unrelated clutches only when associated with their own

**DOI:** 10.1038/s41598-020-80771-7

**Published:** 2021-01-14

**Authors:** Anyelet Valencia-Aguilar, Juan M. Guayasamin, Cynthia P. A. Prado

**Affiliations:** 1 Pós-Graduação em Ecologia, Evolução e Biodiversidade, Instituto de Biociências, Universidade Estadual Paulista “Júlio de Mesquita Filho”, Rio Claro, São Paulo 13506-900 Brazil; 2 Laboratorio de Biología Evolutiva, Colegio de Ciencias Biológicas y Ambientales COCIBA, Instituto Biósfera USFQ, Universidad San Francisco de Quito USFQ, Cumbayá, Ecuador; 3 Departamento de Morfologia e Fisiologia Animal, Faculdade de Ciências Agrárias e Veterinárias, Universidade Estadual Paulista “Júlio de Mesquita Filho”, Jaboticabal, São Paulo 14884-900 Brazil; 4 Department of Biology, University of North Carolina at Chapel Hill, Chapel Hill, NC 27599-3280 USA

**Keywords:** Zoology, Animal behaviour, Herpetology, Ecology, Behavioural ecology

## Abstract

Parental care is costly, thus theory predicts that parents should avoid caring for unrelated offspring. However, alloparenting has been reported in many taxa because it may increase the caregiver mating success or offspring survival. We experimentally investigated the existence of allopaternal care in two glassfrog species, *Hyalinobatrachium chirripoi* and *Centrolene peristicta*, and discussed possible costs and benefits. Males mated with multiple females and cared for clutches, while continued to call. In the field, we randomly placed unrelated clutches in the territory of males already caring for their clutches and in the territory of non-attending males. Attending males adopted unrelated clutches, whereas non-attending males abandoned their territories. Once males adopted unrelated offspring, they cared for all clutches in a similar frequency and gained new clutches. Alloparenting was context-dependent, as only males already caring for their clutches adopted unrelated ones. We suggest that steroid hormonal levels might mediate the adoption of unrelated offspring by attending males. Additionally, our results suggest that males do not directly discriminate between related and unrelated offspring. Alloparenting has been widely investigated in different vertebrates, except for amphibians. Thus, our study sheds light on the roles of alloparenting for offspring survival and mating success in this group.

## Introduction

Parental care involves any parental behavior that increases both parent and offspring fitness, with potential costs to the caregivers^1^. Because the amount of care provided can decrease parental survival and future reproduction^2^, parents should avoid caring for unrelated offspring to save resources for present or future reproductive events^3^. Indeed, a positive correlation between offspring relatedness and care assistance has been reported for some species of birds^4^, fishes^5^ and arthropods^6^. Then, if parental care is beneficial only when directed toward genetic descendants^2^, why do parents sometimes care for unrelated offspring? It has been proposed that alloparenting is a misdirected parental care that result from intraspecific clutch piracy^7,8^ or brood parasitism^9,10^, such as sneak fertilization by younger or smaller males^7,11^. However, in some species parents care for unrelated offspring because of direct benefits, such as higher survivorship of their own offspring or higher mating success^7^. There is a strong selection on females to choose males that provide direct benefits, such as nest sites, territories and parental care, which improve females and offspring fitness^12,13^. In species with paternal care, it is expected that females take into account not only traits that indicate the genetic quality of potential partners, but also the quality of offspring assistance provided by the male^1,14,15^. In fact, in some animal groups, females prefer to mate and spawn in the territory of males already associated with clutches^16,17^. Thus, males may use clutch adoption as part of a reproductive strategy to attract mates^18,19^.

Anurans show elaborate and conspicuous social and reproductive behaviors, making them a natural model system to investigate mating and parental care^20,21^. In the glassfrog family Centrolenidae, males of some species of the genera *Hyalinobatrachium* and *Centrolene* attend clutches deposited on the undersides of leaves above water^22^. A study based on paternity analysis and behavioral observations found that males of the Amazonian species *Hyalinobatrachium cappellei* benefit from paternal care, i.e., males’ chances of mating increased with the number of attended clutches^19^. Moreover, some males remained close to unrelated offspring and were able to attract females and gain their own clutches^19^. Nonetheless, to date, very few studies have investigated the existence of alloparenting in anurans and the costs and benefits of this behavior in the group remains unclear^23,24^.

Herein, we used behavioral observations and clutch translocation experiments to investigate allopaternal care in *Centrolene peristicta* and *Hyalinobatrachium chirripoi*. We compared both care frequency and condition of the clutches experimentally placed in the territories of males already caring for their clutches (attending males) and males without clutches (non-attending males). Under a scenario where males do not exhibit alloparental care, we predict that attending males will care only for their own clutches, while non-attending males will remain close to the unrelated clutch or nearby to attract females, but without caring for them. In species where male alloparenting has evolved, although both related and unrelated offspring may serve for female attraction, only related offspring contributes directly to male’s fitness and should be more valuable for males than unrelated ones^7,18,19^. Thus, if males adopt unrelated clutches, we expect that they will invest less time and energy caring for them, compared to the investment directed towards their own clutches. Parental care decisions can also be influenced by territorial signals^23,24^ because males may use contextual cues (e.g., spatial location, quality of surrounding resources) to recognize their offspring^25,26^, ignoring, abandoning or consuming their own clutches when detecting territory changes^23,24^. On that basis, we experimentally modified and relocated the territories of attending males of *C. peristicta* and *H. chirripoi* to investigate the importance of spatial and territorial cues in the recognition of clutches by males. We expect that males will abandon their clutches after relocation of the territories.

## Results

Males of *Hyalinobatrachium chirripoi* and *Centrolene peristicta* mated with multiple females and cared for clutches in their territories, while continued calling to attract mates. Attending males of *C. peristicta* (*N* = 10) cared for up to two clutches, with a total number of eggs per clutch varying from 19 to 33 (*Ẋ* = 24.05, *sd* =  ± 2.87 eggs, *N* = 36 clutches), and spent on average 27.8% (*sd* =  ± 16.5, *N* = 10) of their time assisting the embryos. Likewise, males of *H. chirripoi* (*N* = 10) cared for up to three clutches, with a total number of eggs per clutch from 58 to 71 (*Ẋ* = 62, *sd* =  ± 5.7 eggs, *N* = 20 clutches), and spent on average 21.9% (*sd* =  ± 9.2, *N* = 10) of their time assisting the embryos.

**Figure 1:**
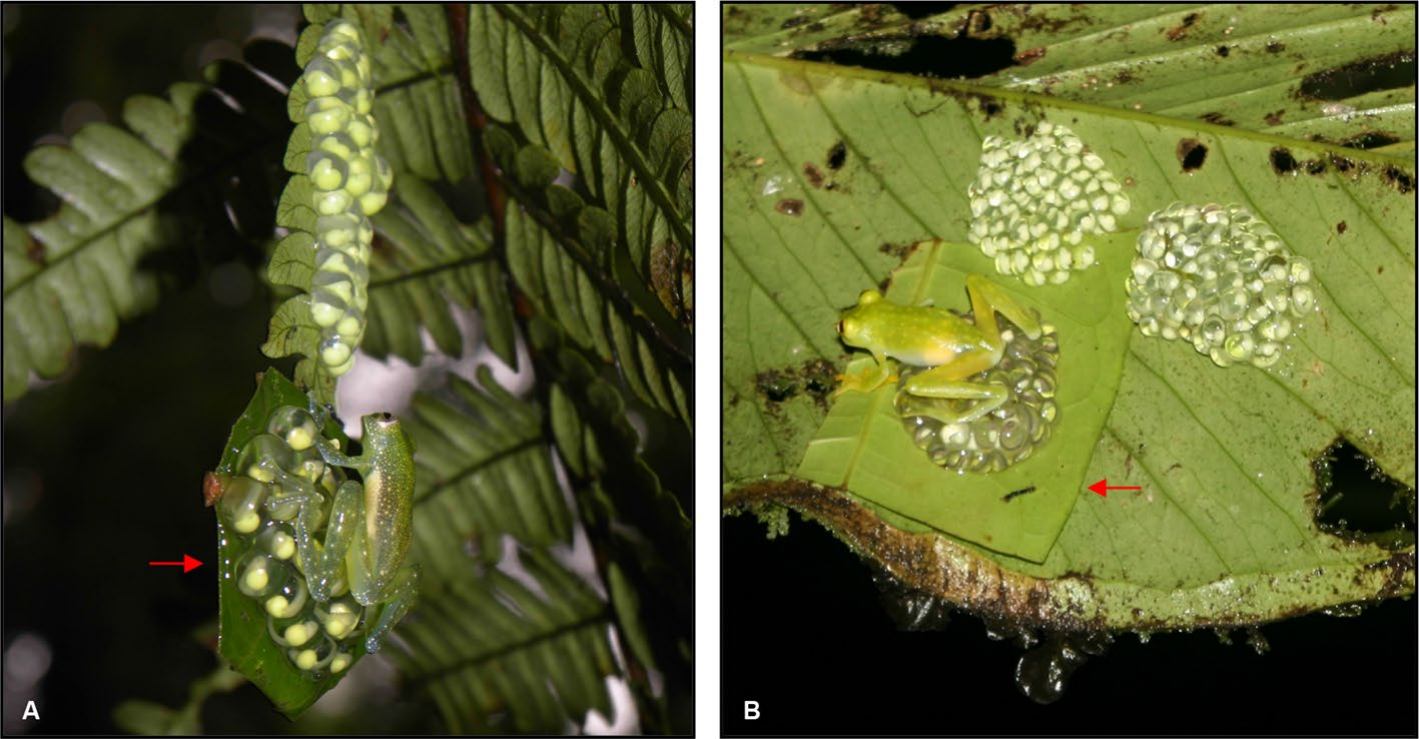
Glassfrog males caring for related and unrelated clutches at different developmental stages. Unrelated clutches were carefully stitched with thread and needle. (**A**) Male of *Centrolene peristicta* caring for an unrelated clutch (pointed by arrow) placed close to his own clutches. (**B**) Male of *Hyalinobatrachium chirripoi* caring for an unrelated clutch (pointed by arrow) placed in his territory. Note that a new clutch (top left) was laid partially on the stitched leaf, where the unrelated clutch was experimentally placed. Anyelet Valencia Aguilar took the photos during the fieldworks in Ecuador.

**Figure 2:**
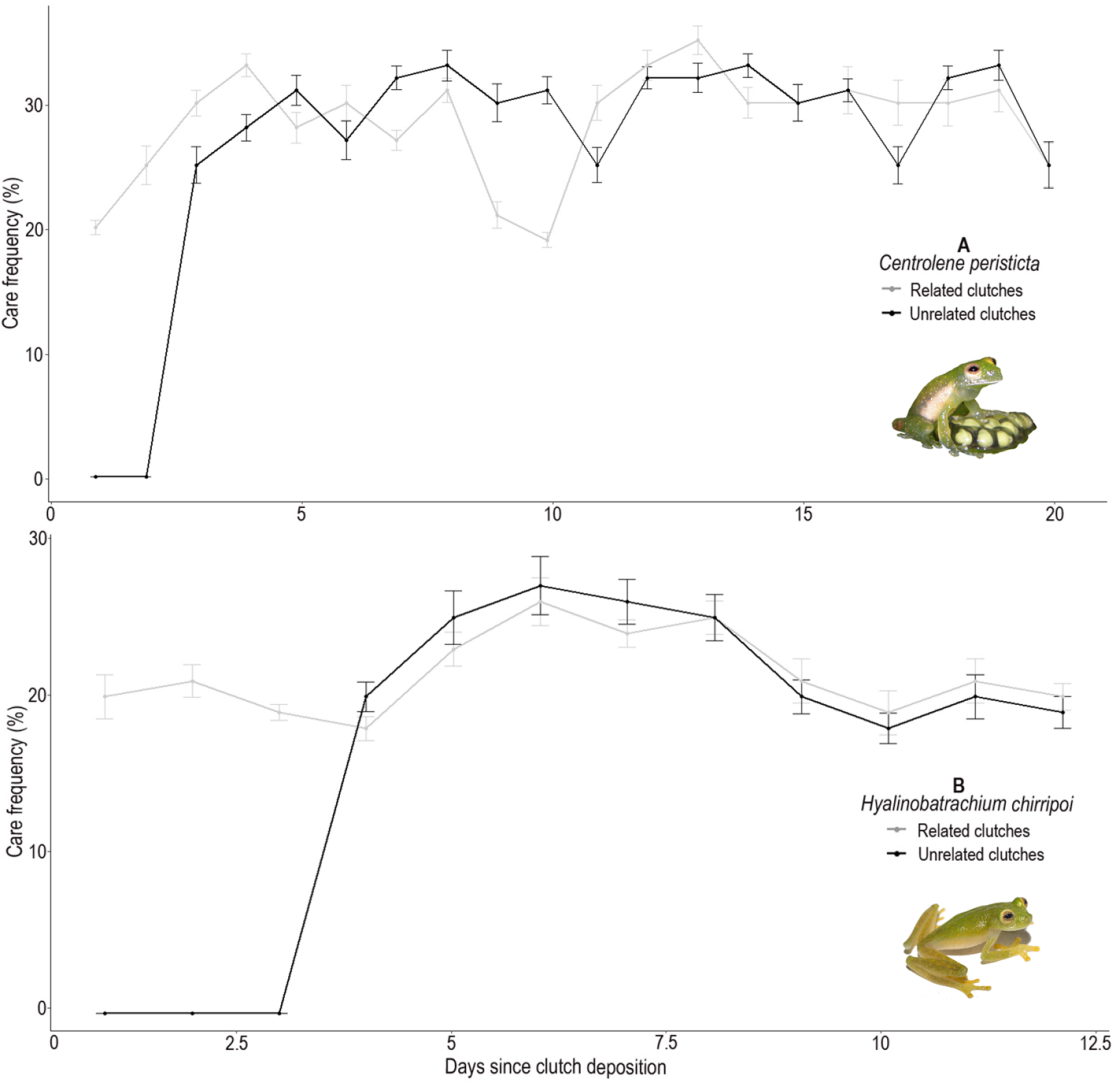
Clutch attendance frequency in two glassfrog species during clutch adoption experiments. (**A**) Time length (days) and care frequency for related and unrelated clutches observed for six males of *Centrolene peristicta* (Photo: Anyelet Valencia Aguilar). (**B**) Time length (days) and care frequency for related and unrelated clutches observed for six males of *Hyalinobatrachium chirripoi* (Photo: Malki Bustos). In (**A**,**B**), each dot represents the mean ± SE for all observed males.

All attending males of *C. peristicta* (*N* = 6) cared for the unrelated clutches experimentally placed in their territories, accepting them as their own clutches (Fig. 1A, Table 1, Supplementary Video S1). Care behaviors included hydrating and handling the embryos to prevent fungus infection. In *H. chirripoi*, half of the attending males (*N* = 3/6) cared for the unrelated clutches placed in their territories (Fig. 1B, Table 1, Supplementary Video S1). Of the remaining three unrelated clutches (each one placed in a different territory), the embryos of one died dehydrated, so we concluded that the male did not provide care. Although we did not observe males hydrating the other two clutches, we observed them touching the unrelated embryos, which developed into larvae and hatched as well as each male’s own embryos (Table 1). Once males of both species noticed the presence of the introduced clutches, they remained between 10 and 20 min close to the new clutch, only observing it; they repeated this behavior three to five times per night after hydrating their own clutches. It took between two to three days for the males to start attending the unrelated clutches (Fig. 2A,B). However, contrary to our expectations, once males started attending, they spent the same proportion of time caring for related and unrelated embryos (*C. peristicta*: *Ẋ* = 28.50%*, sd* = 3.33 for related; *Ẋ* = 27.83%*, sd* = 3.97 for unrelated, *t* test: *t*_28.89_ = 0.45, *p* = 0.60; *H. chirripoi*: *Ẋ* = 21.16%*, sd* = 2.13 for related; *Ẋ* = 20.33%*, sd* = 1.75 for unrelated, *t* test: *t*_20.98_ = 0.57, *p* = 0.15; Fig. 2), and some males were able to acquire new clutches (Table 1). Males of both glassfrog species that were not caring for their embryos (non-attending males) abandoned their territories when unrelated clutches were placed close to them. Once these males noticed the presence of the clutch, they slowly approached and touched (*N* = 2 males of *H. chirripoi*) or observed it (*N* = 4 males of *H. chirripoi*; *N* = 6 males of *C. peristicta*) for a few minutes before moving away. Even more interesting, males moved away few meters from their original location (*C. peristicta*: 3–5 m, *N* = 6; *H. chirripoi*: 4–6 m, *N* = 6) and established a new territory, where some of them were able to attract females and gained their own clutches (*C. peristicta N* = 3 males; *H. chirripoi N* = 2 males).

**Table 1:** Parental care and hatching success for unrelated and related clutches of *Centrolene peristicta* and *Hyalinobatrachium chirripoi*.

Species	Male	Related clutch	Unrelated clutch	New clutch	Hatching success
*Centrolene peristicta*	1	2	1	0	Both
	2	1	1	0	Both
	3	1	1	0	Both
	4	2	1	1	Both
	5	1	1	0	Both
	6	1	1	0	Both
*Hyalinobatrachium chirripoi*	1	1	1	0	Only one related
	2	1	1	0	Both
	3	1	1	0	Both
	4	1	1	1	Both
	5	1	1	1	Both
	6	2	1	0	Both

Male = code for each male monitored in the field; Related clutch = number of clutches that each male was caring for before the clutch introduction experiment; Unrelated clutch = number of clutches placed in each male’s territory; New clutch = number of clutches that each male acquired after unrelated clutch introduction; Hatching success = related and unrelated clutches that hatched after experiment.

Regarding the territory relocation experiment, as we expected, all males in the control group (*N* = 3 males of *H. chirripoi*; *N* = 3 males of *C. peristicta*) remained in their territories despite the changes, caring for their clutches until larvae hatched. As for the experimental group, after territory relocation, half of *H. chirripoi* males (3/6) abandoned the clutches, while all males (6/6) of *C. peristicta* remained and cared for the clutches until larvae hatched. We assume that the males of *H. chirripoi* that abandoned their territories after the experiment did so due to perturbation (some shaking because of the slippery rocks in the stream) during the relocation of the territory (males were on the branch while being moved). Despite this, the other 50% of the males of *H. chirripoi* remained in their territories after relocation and exhibited the same care behavior that we observed in attending males of the control group. The time spent caring for the clutches after territory relocation was not different compared to assistance time provided by males of the control group (*C. peristicta t* test: *t*_20.98_ = 01.42, *p* = 0.15; *H. chirripoi t* test: *t*_17.91_ = 0.62, *p* = 0.32).

## Discussion

Overall, we found that parental care behavior in *Centrolene peristicta* and *Hyalinobatrachium chirripoi* is performed exclusively by males that care for their clutches for over 2 or 3 weeks in their breeding territories, preventing embryo dehydration, fungus infection and predation, as observed in previous studies^19,22,27^. Regarding our adoption experiments, attending males of *C. peristicta* and *H. chirripoi* cared for unrelated clutches in their territories. However, the presence of eggs per se did not stimulate parental care behavior in non-attending males (without clutches); after noticing the presence of the unrelated clutches, they abandoned them within a few minutes. As a comparison, in the poison frog *Allobates femoralis*, males and females transported unrelated tadpoles placed on their backs, suggesting that tactile stimulus was enough to induce care behaviors in adults of this species^28^. In contrast, care behaviors were not triggered by external stimuli (unrelated clutches) in non-attending males of *C. peristicta* and *H. chirripoi*, suggesting that other factors like steroid hormonal levels could be regulating care behaviors in these glassfrogs^29,30^. Hormonal changes can lead to or stimulate paternal care behaviors in species in which care involves attendance, carrying or provisioning of the offspring^31–33^. It is assumed that low levels of testosterone favors paternal care via reduction of mating investment and aggressive behavior^34,35^. For example, attending males of the frog *Eleutherodactylus coqui* showed low androgen levels throughout the clutch attendance period^29^. Although we lack data on hormone levels in the two studied glassfrog species, and territorial aggressive behaviors were rarely observed (A. Valencia-Aguilar, pers. obs.), males of both species continued calling and mating during parental care period. Since minimal levels of androgens are necessary for males to call^34,35^, we suggest that attending and non-attending males of *C. peristicta* and *H. chirripoi* have circulating androgens, but in attending males they might be present at lower levels, which could be tested in future research.

Care activities in some fishes^36^, anurans^32^, birds^37^, and mammals^38^ have been associated to an increase in cortisol levels, which plays a central role in regulating reproductive behavior and mobilizing energy reserves for care behaviors^34,39^. In this sense, we suggest that alloparenting in *C. peristicta* and *H. chirripoi* is context-dependent because unrelated clutches were adopted only by males already caring for their own clutches. As in other frog species^29,35^*,* hormonal shifts in males of *C. peristicta* and *H. chirripoi* may also contribute to promote paternal care behaviors and future studies will help to elucidate the role of hormones in the paternal behavior of these glassfrogs. However, independent on what triggers this behavior, adoption of unrelated clutches may be advantageous for alloparents if the fitness benefit offset the costs^7^. Indeed, caring for unrelated clutches could be costly for non-attending males, reducing their own fitness if they do not acquire their own clutches. In another glassfrog species, *H. cappellei,* males already caring for clutches have higher chances of mating and some males remained close to unrelated clutches as a strategy to attract females^19^. We observed a similar pattern in *C. peristica* and *H. chirripoi,* where both non-attending males that called a few meters from the introduced clutches and attending males that cared for unrelated clutches acquired new clutches after a couple of days. These observations raise the question whether males of these glassfrog species are also using unrelated clutches to increase their attractiveness and consequently mating success. Thus, it is necessary to determine if alloparenting in *C. peristicta* and *H. chirripoi* has no extra costs^40,41^ and if males really benefit in terms of offspring survival^7^ and mating success^42^.

Attending males did not accept the unrelated clutches immediately; instead, they spent a couple of days observing the embryos without caring for them, suggesting that males were able to notice the presence of the new clutch. Therefore, if males detected the presence of the foreign clutch, why they did not decrease care frequency or abandon the territory after been “experimentally cuckolded”? Wisenden^7^ pointed out that many parents do not discriminate between related and unrelated offspring (see discussion below), because either the cost of attending is very low or the cost of eliminating or abandoning them is too high^43,44^. As in other glassfrogs^45–50^, field observations showed that unattended clutches of the studied species died by dehydration, parasitism or predation^50^ (Valencia-Aguilar, pers.obs.). Thus, for *C. peristicta* and *H. chirripoi*, the cost of adopting clutches may be lower than the cost of abandoning them. Indeed, parental care activities do not constrain males from attracting females and foraging^45^ (this study). Additionally, by adopting unrelated clutches, male’s own clutches may benefit from the dilution effect in cases of predation^51,52^. Glassfrog clutches may be preyed by invertebrates (e.g. katydids, spiders) or even snakes^45^, thus an increase in the number of clutches may reduce males´ offspring chances of being preyed^53^. Another explanation for alloparenting could be that, by caring for both related and unrelated clutches, males may avoid potential costs of abandoning their own offspring by mistake. If a male interrupts offspring assistance, he will waste time and energy allocated in parental and mating effort^1,2,44^. Finding a new mate can be difficult for males of both species due to temporal variation in population density and female ‘time out’ mating. The ‘time out’ required to recover from a mating event was estimated to be around two weeks for females of *H. cappellei*^19^. Hence, by losing both care and mating investment as a result of desertion^1,8^, a male will have to invest more to establish a new territory and attract other females. Consequently, attending males may adopt unrelated clutches because alloparenting would not reduce their present or future reproductive success^8^, as we observed that males continued acquiring clutches.

Contrary to our expectations, males of *C. peristicta* and *H. chirripoi* continued caring for their clutches after we have relocated the territory (the entire branch)*.* In an exploratory experiment, we monitored some non-attending males until they acquired one clutch and then we removed the clutch after 4 days of attendance, placing it again close to the corresponding father two days later. Contrary to what we expected, males of both species (*H. chirripoi N* = 1; *C. peristicta N* = 2) abandoned their territories, even when their own clutches were placed back on the same leaf, although in a different position within the territory. Previous studies on dendrobatid frogs that provide parental care suggest that parents are not able to directly discriminate between related and unrelated offspring but use indirect cues, discriminating their location, i.e., offspring presence and position within the territory^23,24^. We could not test whether males of *C. peristicta* and *H. chirripoi* were able to directly differentiate (e.g. via tactile, chemical) between related and unrelated clutches, but based on our observations and experiments, we hypothesize that clutch location inside the territory could trigger parental behavior and alloparenting in these glassfrogs. Further studies should also explore the influence of chemical, visual, or tactile cues for kin recognition in glassfrogs, as observed in some poison frogs^24,54^ and tree frogs^55^.

To conclude, here we showed that males of *C. peristicta* and *H. chirripoi* provide alloparental care when we experimentally introduced an unrelated clutch. However, male decision to provide allocare was context-dependent, likely influenced by hormonal levels, i.e., only attending males adopted clutches, probably to avoid risks to his own clutches in case of desertion and/or to increase mating success. Moreover, parental decision making, involving a switch between adoption or desertion of clutches, may have evolved in the absence of direct offspring discrimination cues (e.g., tactile, chemical). Finally, we suggest that alloparenting may be more widespread among anurans than previously thought and further questions regarding costs and benefits of adoption, kin recognition, and mating strategies remain to be investigated in this group. Alloparental care has been mostly studied in birds and mammals, mainly in species with complex social behaviors^56,57^. Thus, our study presents a novel research avenue on the costs and benefits of adoption in vertebrates with different ecological and evolutionary contexts^24,58,59^.

## Methods

### Study site and parental care behavior

Observations and behavioral experiments were carried out in the Reserves Itapoa (December/2018, 0° 07′ 22.2′′ N, 79° 16′ 16.2′′ W), Canandé (January/2019, 0° 31′ 18.6′′ N, 79° 08′ 09.8′′ W) and Las Gralarias (February–March/2019, 0° 00′ 33′′ S, 78° 44′ 15′′ W), in the Esmeraldas and Pichincha provinces of Ecuador, respectively. We monitored one population of *Centrolene peristicta* (*N* = 10 males; Las Gralarias reserve) and two of *Hyalinobatrachium chirripoi* (*N* = 10 males; Itapoa and Canandé reserves) for an entire breeding season to collect data on mating system, as well as paternal care activities. Males were located and monitored during the day and night with headlamps (red-light) through visual and acoustic searches. Focal observations were made at 1.0 m of distance from each male during 20–40 min during the day and at night (see Supplementary Table S2), when individuals were active (twice or three times per night). During observations, we recorded male activity (calling, mating, aggressive or defensive behaviors against potential predators or competitors), male position relative to embryos (next, touching, sitting on), clutch attendance frequency, and egg/embryo clutch condition (parasitism, predation, embryonic development). Clutch attendance frequency was estimated as the proportion of time that each male spent handling, hydrating or protecting the clutch relative to the total hours of observation during the day, between 09:00 and 13:00, and at night, between 19:00 and 02:00. Then, we estimated time budget for each male by summing occurrences of a given activity across days and divided by the total days of observation. These data allowed us to determine how often males cared for their embryos (hydrate, touch or protect them), which was used as a base line for behavioral experiments and comparison. Each male was used just once in the behavioral experiments (see below). A high-resolution digital video camera recorder in infrared night-shot mode (Sony Handycam AVCHD) was used to visual monitor both behaviors and experiments. The video camera was held in the territory of each male (close enough for monitoring without disturbance) and activated to record in the night vision function to allow observations without flashlights.

### Clutch adoption experiment

Before starting the experiments, we monitored a group of males of both *C. peristica* (*N* = 10) and *H. chirripoi* (*N* = 10) species throughout the entire breeding season for the baseline behavior comparisons (see above). Males of *C. peristicta* (*N* = 12) and *H. chirripoi* (*N* = 12) were randomly assigned to two different treatments: (1) attending males (*N* = 6): individuals already caring for their own clutches, to which one unrelated clutch was experimentally added; (2) non-attending males (*N* = 6): individuals without clutches, to which one unrelated clutch was also experimentally added. All unrelated clutches were obtained from males located 50–80 m away and embryos were at the same developmental stage^60^ and size, in the case of the attending group. In order to move clutches, we cut a piece of the leaf where the clutch was placed and relocated it to a territory of another male, carefully stitching the leaf with thread and needle during the day (Fig. 1), when males were inactive, to minimize disturbance. To compare male’s behavior relative to clutches, we measured the care frequency of both clutch types (related vs. unrelated), which was defined as the proportion of time that an individual spent hydrating, defending or in contact with the embryos (touching them with their body, hands or feet) relative to the length of the observation period. Care frequency was calculated as mentioned above and total observation time is in Supplementary Table S2. We compared care frequency between related and unrelated clutches using Student’s t-test.

### Territory changes and territory relocation experiments

Males used in this experiment were not the same used for the baseline behavioral observations or for the clutch adoption experiment. Attending males were randomly assigned to the control group (*N* = 3 males of each species, due to limited availability of attending males) and the relocated group (*N* = 6 males of each species). In the control group, males’ territories were partially modified to control for handling effects. We cut some of the branches and leaves near the leaf where the male was caring for his clutches. In the relocated group, the entire branch with clutches on the leaves, where the male established its territory, was removed from its original location and transferred to a new one. Based on previous observations, we considered as territory the branch used by males to call, forage, and attend clutches. To examine the importance of spatial location in the care behavior, males were relocated after providing 5 days of care (embryos at stages 19–20 ^60^). To ensure that the new location was different from the original one, we moved males’ territories with their clutches 60 m away, either up or down stream from their original position. Besides location, we also modified the original height of the territories (3–5 m above the stream), placing them between 2 and 3 m above the stream. All changes and relocations of territories were made during the day, when males were inactive, to minimize disturbance. Additionally, relocated branches did not affect embryos’ development as they started to dry after larvae had hatched. The time that males spent in their territories after the relocation was used as the behavioral response. We also observed parental behavior performed by males in both groups and compared care frequency between males in the control group and males in the relocated territory using Student’s t-test. All statistical analyses were performed using the software R 3.1.0 (R Development Core Team 2014).

### Ethical considerations

Field observations and behavioral experiments were performed in accordance with the Association for the Study of Animal Behaviour guidelines and current Ecuadorian legislation. Fieldwork and behavioral experiments were approved by the Ministry of Environment of Ecuador (scientific research number 019-2018-IC-FAU-DNB/MAE).

## Supplementary Information

Supplementary Information.

Supplementary Video S1.
