# Supplementary material for: Alloparental care in glassfrogs: males care for unrelated clutches only when associated with their own
Source: Sci Rep. 2021 Jan 14;11:1386. doi: 10.1038/s41598-020-80771-7 (PMC7809452; doi:10.1038/s41598-020-80771-7)
Supplement: Supplementary file 1 — Supplementary Information. [file 41598_2020_80771_MOESM1_ESM.doc]

**Supplementary material for:**

**Alloparental care in glassfrogs: males care for unrelated clutches only when associated with their own**

Anyelet Valencia-Aguilar, Juan M. Guayasamin, Cynthia P.A. Prado

**Supplementary video S1.** Male of *Centrolene peristica* caring for unrelated and related clutches in his territory.

**Supplementary Table S2**. Field observations and experiments with males of *Centrolene peristica* and *Hyalinobatrachium chirripoi* in Ecuador*.* Time (days and minutes) that each male was observed in the field during this study.

| **Species** | **Experiment** | **Male** | ***N* days of obs.** | **Observed time (min)** |
| --- | --- | --- | --- | --- |
| *Centrolene peristicta* | Control (Behavioral base line) | 1 | 27 | 510 |
|  |  | 2 | 23 | 400 |
|  |  | 3 | 26 | 500 |
|  |  | 4 | 23 | 405 |
|  |  | 5 | 22 | 400 |
|  |  | 6 | 20 | 380 |
|  |  | 7 | 28 | 530 |
|  |  | 8 | 27 | 520 |
|  |  | 9 | 25 | 470 |
|  |  | 10 | 28 | 515 |
|  | Non-attending male + unrelated clutch | 1 | 15 | 310 |
|  |  | 2 | 16 | 380 |
|  |  | 3 | 16 | 400 |
|  |  | 4 | 15 | 290 |
|  |  | 5 | 17 | 350 |
|  |  | 6 | 16 | 320 |
|  | Attending male + unrelated clutch | 1 | 26 | 650 |
|  |  | 2 | 23 | 520 |
|  |  | 3 | 24 | 580 |
|  |  | 4 | 24 | 590 |
|  |  | 5 | 26 | 620 |
|  |  | 6 | 20 | 505 |
|  | Territory change | 1 | 30 | 680 |
|  |  | 2 | 30 | 710 |
|  |  | 3 | 27 | 680 |
|  |  | 4 | 28 | 600 |
|  |  | 5 | 31 | 700 |
|  |  | 6 | 30 | 705 |
| *Hyalinobatrachium chirripoi* | Control (Behavioral base line) | 1 | 15 | 600 |
|  |  | 2 | 11 | 440 |
|  |  | 3 | 14 | 560 |
|  |  | 4 | 15 | 600 |
|  |  | 5 | 12 | 480 |
|  |  | 6 | 11 | 440 |
|  |  | 7 | 14 | 560 |
|  |  | 8 | 16 | 640 |
|  |  | 9 | 15 | 600 |
|  |  | 10 | 16 | 640 |
|  | Non-attending male + unrelated clutch | 1 | 10 | 500 |
|  |  | 2 | 8 | 400 |
|  |  | 3 | 9 | 450 |
|  |  | 4 | 10 | 500 |
|  |  | 5 | 9 | 450 |
|  |  | 6 | 8 | 400 |
|  | Attending male + unrelated clutch | 1 | 15 | 750 |
|  |  | 2 | 14 | 700 |
|  |  | 3 | 14 | 710 |
|  |  | 4 | 13 | 650 |
|  |  | 5 | 11 | 600 |
|  |  | 6 | 11 | 605 |
|  | Territory change | 1 | 7 | 420 |
|  |  | 2 | 8 | 480 |
|  |  | 3 | 8 | 480 |
|  |  | 4 | 10 | 510 |
|  |  | 5 | 9 | 510 |
|  |  | 6 | 8 | 480 |
